# Results of a two-phased clinical study evaluating a new multiband mucosectomy device for early Barrett’s neoplasia: a randomized pre-esophagectomy trial and a pilot therapeutic pilot study

**DOI:** 10.1007/s00464-018-6582-5

**Published:** 2018-11-19

**Authors:** K. Belghazi, D. W. Schölvinck, M. I. van Berge Henegouwen, S. S. Gisbertz, B. L. Weusten, S. L. Meijer, J. J. Bergman, R. E. Pouw

**Affiliations:** 1 Department of Gastroenterology and Hepatology, Amsterdam UMC, University of Amsterdam, Meibergdreef 9, 1105 AZ Amsterdam, The Netherlands; 2 Department of Gastroenterology and Hepatology, St. Antonius Hospital, Nieuwegein, The Netherlands; 3 Department of Surgery, Amsterdam UMC, University of Amsterdam, Amsterdam, The Netherlands; 4 Department of Pathology, Amsterdam UMC, University of Amsterdam, Amsterdam, The Netherlands

**Keywords:** Endoscopic mucosal resection, Multiband mucosectomy, Barrett’s esophagus, High-grade dysplasia, Early cancer

## Abstract

**Background:**

Multiband mucosectomy (MBM) is the preferred technique for piecemeal resection of early neoplastic lesions in Barrett’s esophagus (BE). The currently most widely used device for MBM is the Duette device. Recently, the Captivator EMR device has come available which might have practical advantages over the Duette device.

**Methods:**

*Phase I* was a randomized pre-esophagectomy trial with a non-inferiority design aiming to compare EMR specimens obtained with the Captivator and the Duette device. Primary outcome: max diameter of the EMR specimens, secondary outcomes: min diameter, max thickness of the EMR specimens and resected submucosal stroma. *Phase II* were clinical pilot cases aiming to evaluate the feasibility of EMR using the Captivator device. Primary outcome was the successful EMR rate and secondary outcomes included procedure time and adverse events.

**Results:**

*Phase I*: 24 EMR specimens (12 pairs) were obtained from six patients. The median max diameter of EMR specimens obtained with the Captivator device was 16 mm [IQR 12–21] versus 18 mm [IQR 13–23] for the Duette device. Non-inferiority of the max diameter of the Captivator specimens could not be demonstrated (median difference 1 mm, 95% CI − 3.26 to + 5.26). However, when using paired analysis, no significant difference was found (*p* 0.573). In addition, no statistically significant differences were found in the min diameter, max thickness of EMR specimens, and max thickness of resected submucosal stroma. *Phase II*: 5 BE patients with early neoplastic lesions were included. Successful EMR was achieved in 100%. Median procedure time was 33 min (IQR 25–39). One patient developed transient dysphagia, without signs of stenosis on endoscopy.

**Conclusions:**

EMR of early Barrett’s neoplasia using the Captivator device is comparable to Duette EMR when looking at size of resected specimens. In the first patients, EMR using the Captivator was feasible, resulting in successful resection without acute adverse events.

Endoscopic mucosal resection (EMR) is the cornerstone of endoscopic therapy for early Barrett’s neoplasia (i.e., high grade dysplasia [HGD] or early carcinoma [EC]). The aim of EMR is twofold since it serves as a diagnostic tool by providing a specimen for accurate histopathological assessment, which guides further management, and it may cure by removing the neoplastic lesion.

Multiband mucosectomy (MBM) is a widely used EMR technique, which uses a modified variceal band ligator. The MBM device consists of a control handle that is attached to the proximal end of the endoscope and which is connected to a plastic cap with six rubber bands attached to the tip of the endoscope via a trigger wire. By suctioning a mucosal lesion into the cap and then releasing a rubber band, a pseudopolyp is created that can be resected using a hexagonal electrocautery snare [1–4]. Contrary to the EMR-cap technique, no submucosal lifting or prelooping of the snare in the cap is required for MBM. A randomized clinical trial comparing MBM to EMR-cap showed that MBM is as effective and safe as the EMR-cap technique in piecemeal resection of early neoplastic lesions, but is more user-friendly. Therefore, MBM is preferred over EMR-cap for piecemeal resection of early Barrett’s neoplasia [5–7].

The Duette device (Duette™, Cook, Limerick, Ireland) is currently the most widely used MBM device and it has been proven to be effective and safe [6]. Recently, a new MBM-device (Captivator™ EMR, Boston Scientific Corporation, Natick, MA, USA) has come available, which has several potential advantages over the Duette device. The Captivator device was developed to improve endoscopic view through the plastic cap attached to the tip of the endoscope, by moving the rubber bands from the distal to the proximal end of the plastic cap. Furthermore, the Captivator device uses a metal trigger wire instead of a fibrous trigger wire. Whereas the fibrous wire saturates with fluids in the working channel of the endoscope, leading to swelling of the wire and reduction of the free space in the working channel, the metal wire is significantly thinner and does not swell when in contact with fluids. These potential advantages were supported by an in vitro study comparing the Duette and the Captivator device. That study showed that the Captivator device leads to improved endoscopic visualisation, easier passage of endoscopic accessories through the working channel, and marginally improved suction power when compared to the Duette device [8]. However, in order to be of added value in clinical practice, the performance of the Captivator device should also be equal to the Duette device. Therefore, in *phase I* of this study, we aimed to assess whether the size of EMR specimens obtained with the Captivator and Duette device are comparable. Furthermore, because the Captivator device has not yet been investigated in a clinical setting, we aimed to evaluate the feasibility of EMR using the Captivator device in BE patients with early neoplastic lesions in *phase II*.

## Materials and methods

This single center study conducted in a tertiary referral center for the management of early Barrett’s neoplasia (Amsterdam University Medical Center, location AMC Amsterdam, the Netherlands) consisted of two phases, which will be described separately below. *Phase I* was a randomized trial in patients immediately prior to scheduled esophagectomy and *phase II* consisted of 5 therapeutic clinical pilot cases.

### Multiband mucosectomy devices used in this study

The Captivator device (Boston Scientific Corporation, Natick, MA, USA) consists of a plastic control handle; a metal trigger wire; a transparent plastic cap with six rubber bands at the proximal side of the cap; and a 5-Fr stiff hexagonal snare (*ø* 1.8 mm).

The Duette device (Cook Medical, Limerick, Ireland) includes a control handle, a fibrous trigger wire, a transparent plastic cap with six rubber bands mounted on the distal end of the cap, and a 5-Fr hexagonal snare (*ø* 1.7 mm).

In both devices, the control handle is assembled at the proximal end of the working channel. The trigger wire is advanced through the working channel, followed by placement of the cap on the tip of the endoscope with the trip wire in correct position in the endoscopic field.

### Phase I, randomized pre-esophagectomy clinical trial

#### Patient selection

Patients with esophageal cancer scheduled for transthoracic esophagectomy with intrathoracic or cervical anastomosis were screened. An estimation of the amount of available esophageal tissue for EMR was made based on prior endoscopy reports. Patients were considered eligible for participation if EMR was deemed possible on at least one level (± 2 cm) in the esophagus, located at least 2 cm proximal to the cancer and 3 cm below the intended level of the anastomosis. Patients were excluded if they had undergone prior endoscopic treatment of the intended EMR zone, and/or in case of esophageal stenosis or scarring limiting access to the intended EMR zone.

#### Study procedure: pre-esophagectomy EMR

Patients were prepared for surgery according to local hospital guidelines. After the patient was sedated and intubated, a diagnostic endoscope (Olympus GIF HQ-190) was introduced and the area suited for EMR was delineated by placing coagulation markings 3 cm distal to the planned level of the surgical anastomosis and 2 cm proximal to the cancer. EMRs were performed in pairs at the same level within the esophagus: one resection with each device. Areas were marked with coagulation markings at the 3 o’clock and 9 o’clock position at each level. The randomization for each patient consisted of two stages. First, randomization to either the Duette or Captivator device took place by using a sealed opaque envelope followed by assemblage of the assigned device on the endoscope. A second randomization by sealed opaque envelopes was carried out for each pair of marked areas separately to allocate if EMR was performed at 3 or 9 o’clock, and EMRs were then performed at each level. In some procedures, EMR specimens were retrieved immediately after resection and numbered according to the level at which they were resected, to allow paired comparison. In other procedures, EMRs specimens were removed simultaneously after all resections with one device were performed. By placing electrocoagulation markers on the EMR site (one for level one, two for level two, etc), level of resection could be identified for all specimens. Subsequently, the second device was assembled and EMR at the contra-lateral side from the first EMR was performed at each level, after which all specimens were again collected.

After completion of the endoscopy, all EMR specimens were numbered for tracking purposes and pinned down on paraffin prior to fixation in formalin. All specimens were handled by the same research nurse blinded to the allocated resection tool.

#### Outcome parameters

##### Primary outcome

Maximum diameter of the EMR specimens (mm), as measured by a pathologist blinded to the allocated resection technique.

##### Secondary outcomes

1. Minimum diameter of EMR specimens (mm), maximum thickness of EMR specimens (mm), and maximum thickness of resected submucosal stroma (mm).
2. Intra-procedural adverse events.
3. Duration of the EMR procedure per device, defined as the period between the first introduction of the endoscope and the removal of the endoscope.

#### Sample size

It was anticipated, based on unpublished data from prior Boston Scientific in vivo testing in a porcine model, that there would be no difference in the diameter of EMR specimens performed with the Captivator device and the Duette device. Therefore, a non-inferiority design was set up comparing pairs of EMR specimens at identical levels within the esophagus.

A total number of 12 EMR pairs would be required, when using a significance level of 0.05 and a power of 90%, to demonstrate that the maximum diameter of the EMR specimens obtained with the Captivator device was non-inferior to the Duette device. We considered a difference of 3.5 mm in maximum diameter as clinically relevant. Therefore, non-inferiority was defined as ≤ 3.5 mm difference. For the sample size calculation, nQuery Advisor (version 7, Statistical Solutions Ltd, Cork, Ireland) was used.

#### Statistical analysis

Statistical analysis was performed using SPSS statistical software package (version 24; SPSS Inc, Chicago, IL). For descriptive statistics, mean (standard deviation [SD]) was used in case of a normal distribution of variables, and median (interquartile range [IQR]) was used for variables with a skewed distribution. For the primary outcome, the difference between the maximum diameter of the resection specimens obtained with the Captivator and the Duette device was determined for randomized pairs. The median difference and confidence interval for the median difference were calculated based on the method proposed by Bonett and Price [9]. Non-inferiority could be concluded when the upper bound of the confidence interval was lower than the pre-specified non-inferiority margin. Where appropriate, the unpaired *t* test or the Wilcoxon rank sum test were used for unpaired data and the paired *t* test or the Wilcoxon signed rank test were used for paired data.

#### Post-hoc analysis

We performed a post-hoc analysis comparing the surface of the EMR specimens of both devices. The surface of the ellipse was calculated using the following formula: π × (maximum diameter × 0.5) × (minimum diameter × 0.5).

### Phase II, clinical pilot cases

#### Patient selection

Consecutive patients scheduled for EMR were included if they met all following criteria:

- Age 18–80 years.
- BE with a visible lesion (≤ 4 cm longitudinal length and ≤ 50% of the circumference) and biopsy-proven HGD or EC.
- No suspicion of submucosal invasion, based on the macroscopic appearance and/or endosonography, if performed.
- No signs of lymph node and/or distant metastasis on endosonography and CT-scanning of the thorax and abdomen, if performed.

Patients were excluded when any of the following criteria were met:

- Prior endoscopic therapy of the intended treatment zone.
- Presence of esophageal stenosis limiting access to the intended treatment zone.
- Scarring by any cause of the intended treatment zone.

#### Endoscopic resection procedure

Endoscopic procedures were performed with patients under monitored anesthesia care. First the esophagus was carefully inspected using high-definition white light endoscopy and narrow band imaging. The macroscopic abnormality was delineated by placing coagulation markings. After delineation, the lesion was sucked into the cap, and after release of a rubber band, the tissue was removed with the hexagonal snare. After the final resection, the wound edges were inspected to assess macroscopic radicality of the resection. The EMR specimens were retrieved, pinned down on paraffin, preserved in formalin, and sent for histopathological evaluation.

#### Post procedural care

All study patients received PPI 40 mg twice a day. In addition, patients were prescribed ranitidine 300 mg at bedtime and 5 mL sucralfate suspension 3 times daily for a period of 2 weeks after the procedure. All patients were contacted by telephone after 2 days to check for any early adverse events after the EMR procedure. After this telephone call, patients completed the study.

According to our local hospital guidelines, patients were scheduled for gastroscopy and if necessary additional endoscopic therapy after 3 months. All adverse events that occurred within 3 months after the EMR procedure were registered.

#### Outcome parameters

##### Primary outcome

Percentage of successful EMR, defined as resection of the lesion and all delineation markings.

##### Secondary outcomes

1. Device or procedure-related adverse events. Timing of adverse events was defined as: ‘acute’ (during the procedure), ‘early’ (up to 48 h after the procedure), and ‘late’ (> 48 h after the procedure). Severity of adverse events was graded as ‘mild’ (unscheduled hospital admission, hospitalization < 3 days, hemoglobin drop < 3 g/dL, no need for transfusion), ‘moderate’ (hospitalization 4–10 days, ≤ 4 units blood transfusion, need for repeat endoscopic intervention, radiological intervention), ‘severe’ (hospitalization 10 days, intensive care unit admission, need for surgery, > 4 units of blood transfused, in the case of stenosis: > 5 dilations, stent placement, or incision therapy), or fatal (death attributable to the procedure).
2. Total procedure time, defined as the period between the first introduction of the endoscope until final removal of the endoscope.
3. Maximum and minimum diameter of EMR specimens (mm).

#### Sample size and statistical analysis

Because this was a pilot study, no sample size calculation was performed. Only descriptive statistics were used (SPSS statistical software package, version 24, SPSS Inc, Chicago, IL): mean (± SD) was used in case of a normal distribution of variables, and median (IQR) for variables with a skewed distribution.

### Histopathological evaluation

EMR specimens were pinned down on paraffin. After fixation in formalin, maximum and minimum diameter of the specimen were macroscopically measured by the pathologist. Then, specimens were cut into 2–3 mm strips, routinely processed to paraffin blocks, cut into 4 µm slides, and stained with haematoxylin and eosin. All EMR-specimens were reviewed by a local expert GI pathologist (S.M). The revised Vienna classification was used for histological grading of dysplasia [10]. The following histological features were assessed: depth of tumor infiltration with submucosal invasion measured in microns, tumor differentiation grade, presence of lymphovascular invasion, radicality of the deep vertical resection margins, and of lateral resection margins in case of en bloc resection.

In addition, maximum thickness of the specimens and the thickness of the submucosal stroma in the specimens were measured microscopically. The pathologist was blinded for the allocated EMR device.

### Ethical considerations

The ethics committees at the Amsterdam University Medical Center, location AMC Amsterdam reviewed and approved the study protocol and the patient informed consent form. Written informed consent was obtained from all patients prior to inclusion. The trial was registered at http://www.trialregister.nl (Registration Number: NTR5286).

## Results

### Phase I

**Fig. 1:**
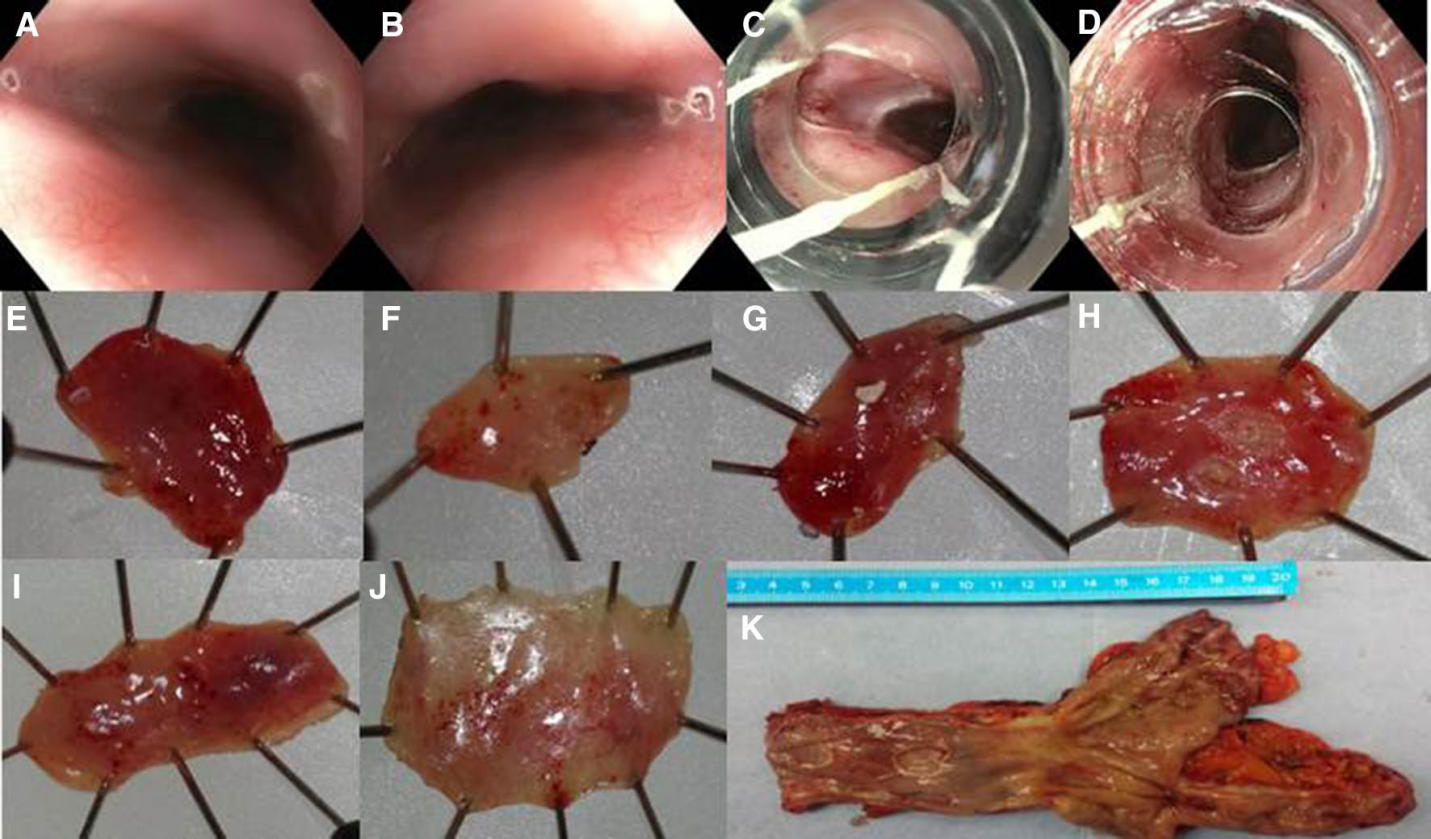
Phase I: six paired endoscopic resections performed on three levels in the esophagus directly prior to the planned esophagectomy. **A, B** Delineation by coagulation markings of the intended resection areas at the 3 o’clock and 9 o’clock position on two levels in the esophagus. **C, D** Endoscopic view through the cap of the Duette device (**C**) and the Captivator device (**D**). **E, J** Three pairs of endoscopic resection specimens pinned down on paraffin: Captivator (**E, G, I**) and Duette (**F, H, J**). **K** Surgical resection specimen showing the endoscopic resection wounds of the six endoscopic resections

Between December 2015 and December 2016, six patients (5 men, median age 63 years [IQR 53–72]) were included. All patients underwent neo-adjuvant chemoradiation therapy followed by transthoracic esophagectomy with intrathoracic anastomosis because of a distal esophageal adenocarcinoma. A total of 24 EMR specimens (12 pairs) were collected with a median of 4 (range 2–6) per patient (Fig. 1).

#### Primary outcome

##### Maximum diameter of EMR specimens

The median maximum diameter of the EMR specimens obtained with the Duette device was 18 mm [IQR 13–23] and 16 mm [IQR 12–21] for the Captivator specimens.

The median difference of the maximum diameter of the paired specimens was 1 mm (95% CI − 3.26 to + 5.26). Since the 95% confidence interval of the median difference exceeds the pre-specified non-inferiority margin, non-inferiority of the maximum diameter of the Captivator EMR specimens could not be demonstrated. However, when comparing the specimens using paired analysis, no significant difference was found in maximum diameter (*p* 0.573) (Table 1).

**Table 1:** Phase II: endoscopic resection specimens obtained with the Captivator and Duette device

	Duette (*n* = 12)	Captivator (*n* = 12)	*p*-value
Median maximum diameter, mm [IQR]	18 [13–23]	16 [12–21]	0.573^a^ 1 mm difference (95% CI − 3.26 to + 5.26)^b^
Median minimum diameter, mm [IQR]	11 [10–13]	13 [10–16]	0.141^a^
Median maximum thickness, mm [IQR]	1.86 [1.47–2.26]	1.96 [1.77–2.10]	0.530^a^
Median maximum thickness submucosa, mm [IQR]	0.55 [0.50–0.73]	0.71 [0.57–0.97]	0.059^a^
Median ellipse ER specimen, mm^2^ [IQR]	148 [100–225]	150 [91–258]	0.583^a^

*ER* endoscopic resection, *IQR* interquartile range, *mm* millimeter, *n* number

^a^Wilcoxon signed rank test

^b^Non-inferiority is demonstrated if the 95% CI of the median difference of the maximum diameter of paired endoscopic resection specimens excludes the pre-specified non-inferiority margin of 3.5 mm

#### Secondary outcomes

##### Minimum diameter, maximum thickness, and maximum thickness of submucosal stroma of EMR specimens

The median minimum diameter and the median maximum thickness of the EMR specimens was 11 mm [IQR 10–13] and 1.86 mm [1.47–2.26] for the Duette device and 13 mm [IQR 10–16] and 1.96 mm [1.77–2.10] for the Captivator device. The amount of resected submucosal stroma was median 0.55 mm [IQR 0.50–0.73] in the EMR specimens obtained with the Duette device and median 0.71 mm [IQR 0.57–0.97] in the Captivator specimens (Table 1).

No significant differences were found for the minimum diameter (*p* 0.141), the maximum thickness of the specimens (*p* 0.530), and the amount of resected submucosal stroma (*p* 0.059).

The median ellipse of the Duette and Captivator specimens was 148 mm^2^ [IQR 100–225] and 150 mm^2^ [IQR 91–258], respectively (*p* 0.583).

##### Intra-procedural adverse events

No adverse events occurred during the EMR procedures using both devices.

##### Procedure time

The procedure time did not differ significantly: median duration with the Duette device was 8 min (IQR 5–18) and 10 min (IQR 6–17) when using the Captivator device (*p*-value 0.916).

### Phase II

**Fig. 2:**
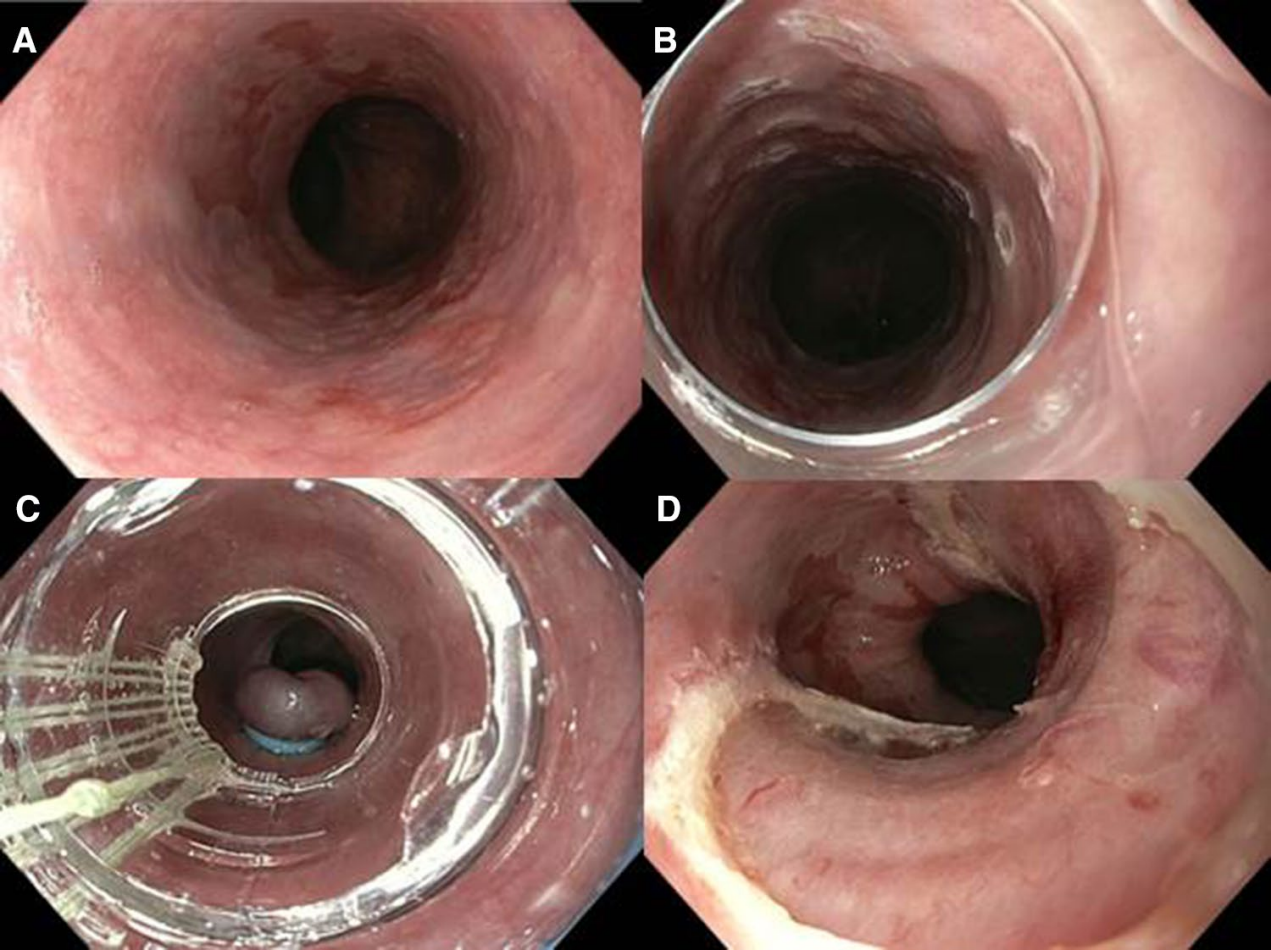
Phase II: endoscopic resection using the Captivator device. **A** a C < 1M5 Barrett’s esophagus with a type 0-IIa–0-IIb lesion of approximately 20 mm at the 5 o’clock position. **B** The lesion (in this image at the 1 o’clock position) is delineated by placing coagulation markings around the lesion. **C** Endoscopic view through the plastic cap of the Captivator device showing the pseudopolyp that is created by suctioning the lesion into the cap and releasing a rubber band. **D** Endoscopic resection wound directly after the successful endoscopic resection

We included 5 patients (4 men) with a median age of 67 years (IQR 56–74) between July and August 2015. The median Barrett’s length was C1 (IQR 0–5) M5 (IQR 3–8). In all patients, a focal lesion containing HGD upon prior biopsies was removed using the Captivator EMR device (Fig. 2). Details on lesion characteristics are presented in Table 2. The worst histopathological diagnosis of the EMR specimens was HGD in two patients and mucosal cancer in three patients.

**Table 2:** Phase II: baseline characteristics

Number of patients, *n*	5
Gender, M:F	4:1
Median age, years (IQR)	67 (56–74)
Median Barrett’s length, cm (IQR)	C1 (0–5) M5 (3–8)
Indication for ER	
Cancer	0
High-grade dysplasia in a focal lesion	5
Diffuse high-grade dysplasia	0
Low-grade dysplasia in a focal lesion	0
Median maximum diameter lesion, mm (IQR)	20 (15–40)
Median maximum circumferential extent lesion, % (IQR)	17 (14–25)
Predominant lesion type^a^, n (%)	
Paris 0-Ip	0
Paris 0-Is	0
Paris 0-IIa	2 (40)
Paris 0-IIb	3 (60)
Paris 0-IIc	0

*cm* centimeter, *ER* endoscopic resection, *IQR* interquartile range, *n* number, *mm* millimeter

^a^Lesion type according to the Paris classification

#### Primary outcome

Successful EMR of the lesion and all delineation markings was achieved in 100% of patients with a median of 2 resections (range 1–10) per procedure.

#### Secondary outcomes

##### Procedure time

Median procedure time was 33 min (IQR 25–39).

##### Adverse events

One patient developed a moderate late adverse event. This was a 56-year-old man with a C < 1M5 BE with a 20 mm large lesion that was successfully removed with the Captivator device in 2 pieces. The patient presented with dysphagia 1 month after the EMR, however, no stenosis was seen upon endoscopy and no dilatation was performed.

##### Size of EMR specimens

A total of 16 EMR specimens was retrieved with median 2 (range 1–10) resections per procedure.

The median maximum and minimum diameter of the EMR specimens were 19 mm (IQR 16–22) and 14 mm (IQR 11–15), respectively.

## Discussion

This is the first in vivo study evaluating the Captivator device for EMR of early Barrett’s neoplasia in a clinical setting. A prior in-vitro study showed that the new Captivator device may have advantages over the Duette device regarding the endoscopic visibility, passage of accessories through the working channel, and suction power [8].

In phase I, we hypothesized that the maximum diameter of EMR specimens obtained with the Captivator device would be equal to specimens obtained with the Duette device. We used a non-inferiority design to evaluate whether the Captivator specimens were comparable to the Duette specimens. Non-inferiority was defined as a median difference ≤ 3.5 mm. We found a median difference of 1 mm (95% CI − 3.26 to + 5.26) in the maximum diameter of the specimens. The upper border of the 95% confidence interval exceeds the pre-specified non-inferiority margin. Therefore, non-inferiority cannot be demonstrated. However, one may question the relevance of this conclusion given the wide 95% confidence interval, which also includes zero suggesting that these results are inconclusive. The size of the EMR specimens showed a great variation. The exact cause of this variation is unclear. However, it is not likely that the variation was caused by the Captivator device since the size of the EMR specimens not only varied greatly between the devices (median difference: +1 mm, range − 14 mm to + 6 mm) but also within each device (Captivator: median 16 mm, range 11–32 mm and Duette: 18 mm, range 9–25 mm). No prior study has directly compared the EMR specimens of the Duette and the Capitvator device before. However, data on the individual devices are available, which allows for comparison with the results of phase I of the current study. A prior randomized trial, comparing EMR using the Duette device to the ER-cap technique, showed that Duette specimens have a median maximum diameter of 18 mm (IQR 15–20) [5]. In *phase II* of this study, EMR with the Captivator device resulted in specimens with a median maximum diameter of 19 mm (IQR 16–22). The data from the previously mentioned randomized trial and from *phase II* of the current study showed less variation in specimen size of both devices which also argues against the study device causing the variation. It is more likely that other factors that were present during phase I of this study may have played a role in the varying specimen size. For example, in phase I of this study, endoscopic resection was performed in previously radiated squamous tissue whereas in the previous randomized trial [5] and in phase II of this study, EMR was performed in Barrett’s tissue that had not been treated before. We believe that the different type of tissue that was resected may have played a role in the variation that was observed.

When comparing the EMR specimens from both devices, using a paired analysis of EMRs performed at the same level, no significant difference was found in the maximum diameter. In addition, the minimum diameter, surface area, the maximum thickness of the EMR specimens, and the amount of resected submucosal stroma did not significantly differ between the Captivator and Duette device. Given the comparable outcomes for the Captivator and Duette device in this study and the abovementioned advantages of the Captivator device [8], one may give preference to the Captivator device on theoretical grounds.

In phase II of this study, all 5 patients with early neoplasia were successfully treated with the Captivator device. The median procedure time of 33 min for Captivator EMR was comparable to procedure times reported in literature for Duette procedures (median 34 min) [5].

This study has a number of limitations that need to be addressed. For the sample size calculation in *phase* I, we assumed that the variability of the EMR specimen size would be comparable to what is known in literature for EMR of Barrett’s tissue using the Duette device. In phase I, however, EMRs were performed in squamous mucosa that was treated with chemoradiation. Given the high variability of specimen size in *phase I* of this study, the study may have benefitted from a large sample size. Phase *II* was a pilot study with a limited number of patients. One should, therefore, be careful with drawing conclusions on the efficacy and safety of EMR using the new Captivator device, based on the results of this pre-clinical and pilot study. In addition, the Captivator procedures were performed by two endoscopists (J.B and B.W) with extensive experience in the work-up and endoscopic treatment of early Barrett’s neoplasia.

Further studies on the Captivator device are needed. The safety and efficacy of the Captivator device for resection of early Barrett’s neoplasia is currently being studied in a large prospective multicenter registry (Trial Registration Number NCT02482701).

In conclusion, EMR using the Captivator device is comparable to Duette EMR when looking at size of resected specimens. In the first five patients, EMR using the Captivator resulted in successful resection, without acute adverse events. Additional data on efficacy and safety will need to be demonstrated by a large cohort study.
